# A Delayed Acute Vestibular Syndrome and Diplopia in Ramsay Hunt Syndrome With Absent Facial Nerve Paralysis After Partially Treated Varicella-Zoster Virus (VZV) Oticus

**DOI:** 10.7759/cureus.99653

**Published:** 2025-12-19

**Authors:** Ambuj Bhalla, Zeeshan Zubair, Lisle W Blackbourn, Jorge Kattah

**Affiliations:** 1 Department of Neurology, University of Illinois College of Medicine Peoria, Peoria, USA; 2 College of Medicine, University of Illinois College of Medicine Peoria, Peoria, USA

**Keywords:** acute vestibular condition, cerebello-pontine angle, diplopia, herpes zoster virus, indications for cranial mri, nystagmus, ramsay hunt syndrome, varicella-zoster

## Abstract

Ramsay Hunt syndrome (herpes zoster oticus) is an uncommon neurological complication of varicella-zoster virus (VZV) reactivation caused by inflammation of the geniculate ganglion of cranial nerve VII. While Ramsay Hunt syndrome classically affects cranial nerve VII, concomitant involvement of cranial nerve VIII is well described. The classic triad includes unilateral lower motor neuron facial paralysis, otalgia, and vesicular rash in the auricle or auditory canal. Atypical presentations without these features are recognized and may delay diagnosis.

We report an 82-year-old man with recent herpes zoster oticus who developed acute vestibular syndrome with gait instability, left-beating nystagmus, and vertical diplopia. He had left ear and temporal pain but no facial weakness. Contrast-enhanced MRI showed enhancement of cranial nerves VII and VIII in the cerebellopontine angle without clinical facial motor deficit.

Following sequential antiviral therapy (intravenous acyclovir followed by oral valacyclovir) and corticosteroids, diplopia and nystagmus resolved within one week with substantial gait improvement by four weeks.

This case highlights Ramsay Hunt syndrome presenting with predominant vestibulocochlear dysfunction despite radiographic facial nerve involvement. Prompt antiviral and corticosteroid therapy can prevent permanent sequelae such as sensorineural hearing loss or neurotrophic keratitis. Clinicians should maintain a high index of suspicion for VZV-related cranial polyneuropathy in older adults with acute vestibular syndrome after recent zoster, even in the complete absence of facial palsy.

## Introduction

Ramsay Hunt syndrome, also known as herpes zoster oticus, is a rare late complication of varicella-zoster virus (VZV) reactivation that causes inflammation of the geniculate ganglion of cranial nerve VII [1]. After primary infection (chickenpox), VZV largely remains latent in cranial nerve and dorsal root ganglia [2-5]. Reactivation - which may occur at any age and is not exclusively related to immunocompromise (although advanced age and immunocompromised states increase risk) - typically manifests as a painful vesicular rash in a single dermatome [2]. In very rare cases (<1%), the geniculate ganglion is involved, producing Ramsay Hunt syndrome [3].

Although most herpes zoster cases follow a predictable dermatomal pattern, atypical presentations - particularly those lacking facial weakness despite geniculate involvement - are under-recognized and frequently lead to delayed diagnosis and treatment. Herein, we present an 82-year-old man with Ramsay Hunt syndrome manifesting as acute vestibular syndrome and diplopia in the complete absence of facial nerve paralysis.

## Case presentation

An 82-year-old male with a past medical history of atrial fibrillation (on Apixaban), hypertension, obesity, and recent herpes zoster oticus presented with one week of progressive gait instability, vertical diplopia, and left temporal pain.

Ten days earlier, he developed a painful vesicular rash in the left external ear, concha, and temporal region, diagnosed as herpes zoster oticus and treated with oral valacyclovir plus prednisone. Despite partial pain improvement, he developed marked imbalance with truncal ataxia requiring a walker.

Neurologic examination showed persistent left-beating horizontal nystagmus, positive head-impulse test to the right (consistent with left peripheral vestibular dysfunction), and episodic vertical diplopia suggestive of skew deviation. Facial strength remained normal bilaterally (House-Brackmann grade I). No central deficits were present (normal coordination, speech, sensation); Dix-Hallpike testing was negative.

**Figure 1:**
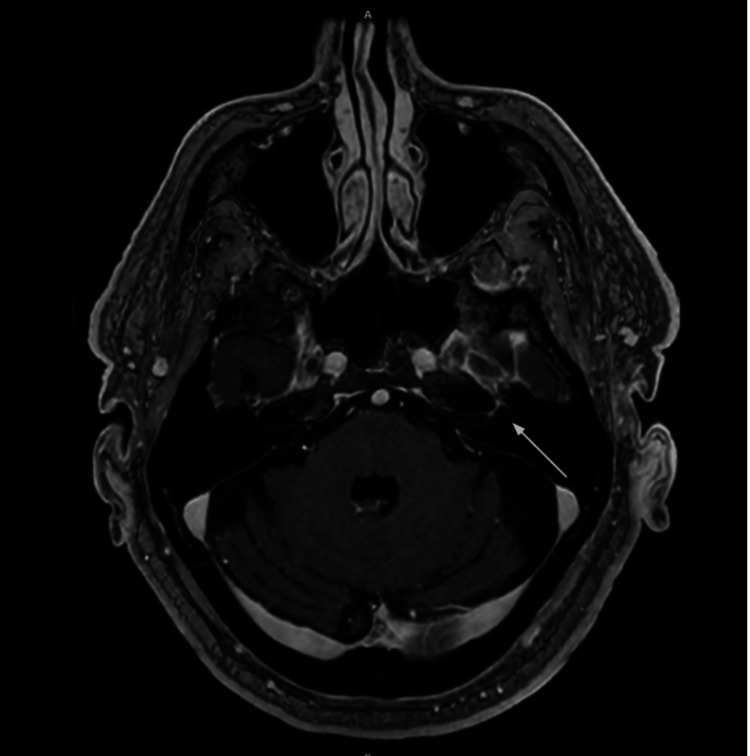
Axial post-contrast MRI demonstrating abnormal enhancement of the left facial nerve (cranial nerve VII) within the labyrinthine segment.

**Figure 2:**
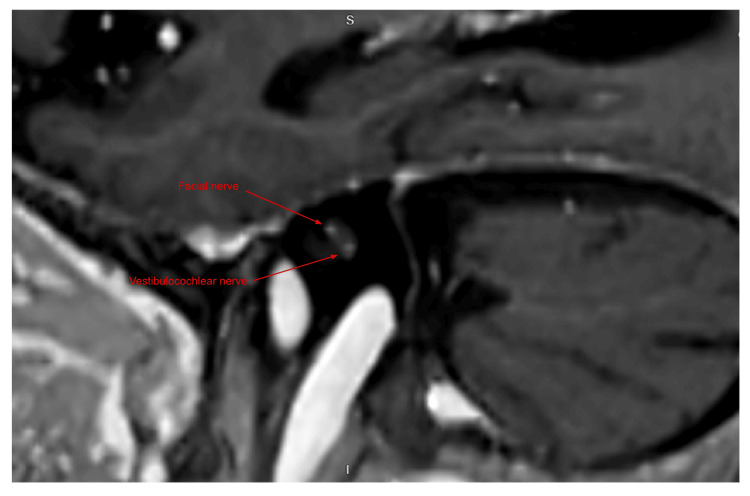
Sagittal T1-weighted post-contrast MRI of the internal auditory canal. The red arrows identify enhancement of the facial nerve (located anterior-superiorly) and the vestibulocochlear nerve (specifically the anterior-inferior cochlear branch), consistent with Ramsay Hunt syndrome.

**Figure 3:**
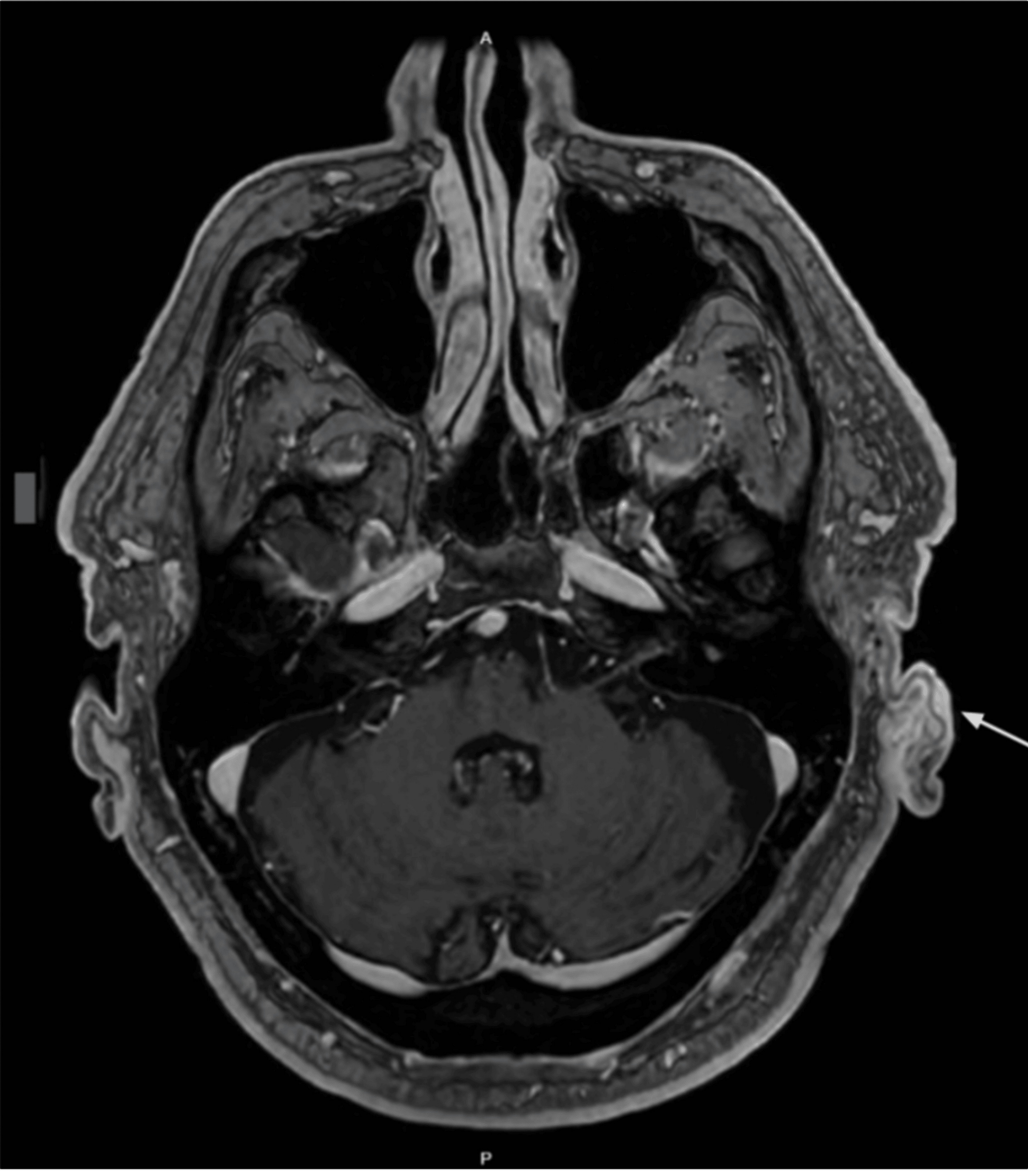
Axial post-contrast MRI demonstrating enhancement of the left external ear and periarticular soft tissues, consistent with active varicella-zoster virus (VZV) reactivation.

Serum VZV IgG was positive (IgM negative), confirming reactivation. Non-contrast brain MRI excluded an acute infarct or hemorrhage. Contrast-enhanced FIESTA sequences demonstrated abnormal enhancement of the left facial (CN VII) and vestibulocochlear (CN VIII) nerves (Figures 1-2) and of the left external ear/soft tissues (Figure 3). A cervical spine MRI, obtained to exclude radiculopathy contributing to gait instability, showed only age-related degenerative changes. Lumbar puncture revealed mild lymphocytic pleocytosis (18 WBCs) with normal protein/glucose; CSF VZV polymerase chain reaction (PCR) was negative. The clinical timeline is summarized in Table 1.

**Table 1:** Clinical timeline of symptom onset and key events.

Day/Week	Key Events
Day 0	Vesicular rash diagnosed; oral valacyclovir + prednisone started
Day 7	Gait instability and diplopia onset
Day 10	Admission; intravenous acyclovir initiated; contrast MRI performed
Day 17	Diplopia/nystagmus resolved
Week 4	Independent ambulation with mild residual pain

He received intravenous acyclovir 10 mg/kg every eight hours for three days, followed immediately by oral valacyclovir 1 g three times daily to complete 14 days of antiviral therapy (sequential regimen), plus a seven-day prednisone taper (starting 60 mg daily). Diplopia and nystagmus resolved within one week; gait improved substantially. At four-week follow-up, he was ambulating independently with mild residual jaw/ear discomfort and no permanent visual or auditory deficit.

## Discussion

This case describes an 82-year-old man who developed acute vestibular syndrome and diplopia 10 days after herpes zoster oticus, with complete sparing of facial motor function despite radiographic enhancement of CN VII and VIII.

Differential considerations, including posterior circulation stroke, multiple sclerosis, and vestibular migraine, were excluded by the subacute onset, absence of central neurologic signs, normal non-contrast MRI, characteristic CN VII/VIII enhancement, and rapid clinical improvement after antiviral/corticosteroid therapy. Specifically, posterior circulation stroke was unlikely due to the absence of vascular risk factors beyond controlled atrial fibrillation (on anticoagulation), lack of focal brainstem signs (e.g., crossed sensory loss, dysarthria, Horner syndrome), normal non-contrast MRI (no diffusion restriction or infarct), and subacute rather than hyperacute onset. Similarly, multiple sclerosis and vestibular migraine were ruled out by the absence of prior episodes, negative CSF oligoclonal bands (if tested), no headache history, and prompt symptom resolution with targeted antiviral/corticosteroid therapy rather than spontaneous fluctuation or steroid response alone.

Ramsay Hunt syndrome occurs in <1 % of zoster cases, with facial palsy present in approximately 95 % [1,6]. However, atypical variants sparing motor function but involving CN VIII are reported in 5-15 % of geniculate-zone reactivations, and vestibular-predominant presentations after zoster oticus are well documented, often mimicking stroke and leading to delayed diagnosis when facial weakness is absent [3,4,7,8]. The superior division of the vestibular nerve lies anatomically closest to the geniculate ganglion, which may explain the disproportionate vestibulocochlear symptoms, whereas facial motor fibers appear to possess greater functional reserve or lower viral affinity in some patients [9]. Negative CSF VZV PCR, as seen here, is common in peripheral cranial nerve syndromes and does not exclude the diagnosis when serology and imaging are supportive [10]. Early intravenous acyclovir plus corticosteroids improves outcomes in elderly patients [2].

The rapid progression despite outpatient oral valacyclovir prompted escalation to sequential intravenous acyclovir followed by oral valacyclovir. This intensification produced swift resolution of diplopia and nystagmus with substantial gait recovery.

Key takeaway

Inadequate initial treatment of herpes zoster oticus can allow viral spread to adjacent cranial nerves. Intravenous acyclovir should be considered early in older patients or those with poor response to oral therapy.

## Conclusions

This case illustrates a clinically partial expression of Ramsay Hunt syndrome - predominant vestibulocochlear dysfunction with complete sparing of facial motor function - despite radiographic involvement of both CN VII and CN VIII. Early recognition and prompt antiviral plus corticosteroid therapy can limit viral spread and prevent permanent sequelae such as sensorineural hearing loss or neurotrophic keratitis. In older adults with recent zoster, clinicians should maintain a high index of suspicion for VZV reactivation even in the absence of facial weakness. Heightened awareness in an aging population is essential to optimize outcomes.
